# Postmarketing Surveillance of Thrombectomy Using a Tron FX Stent Retriever for Large and Medium Vessel Occlusion

**DOI:** 10.3390/jcm14227913

**Published:** 2025-11-07

**Authors:** Hirotoshi Imamura, Nobuyuki Sakai, Masataka Takeuchi, Tsuyoshi Ohta, Nobuyuki Ohara, Yukako Yazawa, Yoshinori Akiyama, Maki Fukuda, Keisuke Imai, Chiaki Sakai, Yasushi Matsumoto, Yuji Matsumaru, Hiroshi Yamagami, Shinichi Yoshimura, Yasushi Ito, Naoya Kuwayama, Tatsuo Kagimura

**Affiliations:** 1 Department of Neurosurgery, National Cerebral and Cardiovascular Center, Suita 564-8565, Japan; 2 Department of Neurovascular Research, Kobe City Medical Center General Hospital, Kobe 650-0047, Japan; 3 Department of Neurosurgery, Seijinkai Shimizu Hospital, Kyoto 615-8237, Japan; 4 Department of Neurosurgery, Seisho Hospital, Odawara 250-0001, Japan; 5 Department of Neurosurgery, Kobe City Medical Center General Hospital, Kobe 650-0047, Japan; 6 Department of Neurology, Kobe City Medical Center General Hospital, Kobe 650-0047, Japan; 7 Department of Stroke Neurology, Kohnan Hospital, Sendai 982-8523, Japan; 8 Department of Neurosurgery, Tenri Hospital, Tenri 632-8552, Japan; 9 Department of Neurosurgery, Kochi Health Sciences Center, Kochi 781-0111, Japan; 10 Department of Neurology and Stroke Treatment, Japanese Red Cross Kyoto Daiichi Hospital, Kyoto 605-0981, Japan; 11 Department of Regulatory Science of Medical Device Development and Innovation, Kyoto University Graduate School of Medicine, Kyoto 606-8303, Japan; 12 Division of Development and Discovery of Interventional Therapy, Tohoku University Hospital, Sendai 980-8574, Japan; 13 Department of Neurosurgery, Institute of Medicine, University of Tsukuba, Tsukuba 305-8575, Japan; 14 Department of Stroke Neurology, National Hospital Organization, Osaka National Hospital, Osaka 540-0006, Japan; 15 Department of Neurosurgery, Hyogo Medical University, Nishinomiya 663-8501, Japan; 16 Department of Neurosurgery, Shinrakuen Hospital, Niigata 950-2038, Japan; 17 Department of Neurosurgery, Japanese Red Cross Toyama Hospital, Toyama 930-0859, Japan; 18 Foundation for Biomedical Research and Innovation at Kobe, Translational Research Center for Medical Innovation, Kobe 650-0047, Japan

**Keywords:** Tron FX stent retriever, medium vessel occlusion, mechanical thrombectomy, postmarketing surveillance

## Abstract

**Background/Objectives**: The Tron FX is a stent retriever thrombectomy device that underwent a clinical trial in Japan in 2016. Its key feature is the availability of a 2/15 mm model designed for medium-vessel occlusion (MeVO). This study reports the results of postmarketing surveillance (PMS) conducted from 2019 to 2020. **Methods**: The PMS included data from 240 patients in whom a Tron FX was used during the first pass at 24 Japanese institutions. Occluded vessels involving the M2 and M3 segments of the middle cerebral artery, anterior cerebral artery, and posterior cerebral artery were classified as MeVO. The recanalization rate, first pass effect (FPE) rate, symptomatic intracranial hemorrhage (sICH) rate, and favorable prognosis rate at 90 days were evaluated. Treatment outcomes were also analyzed for cases in which the device was used after the second pass, excluding those with tandem occlusion, atherothrombotic brain infarction (ATBI), or percutaneous transluminal angioplasty (evaluation-appropriate cases), stratified by MeVO and large vessel occlusion (LVO) and according to Tron FX device size. **Results**: A total of 244 cases were enrolled, of which 218 were evaluation-appropriate. Across all cases, the recanalization rate (modified Thrombolysis in Cerebral Infarction score ≥ 2b) was 70.9%, the FPE rate was 23.4%, the sICH rate was 3.8%, and the proportion of patients with a good prognosis (modified Rankin Scale score 0–2 at 90 days) was 53.1%. Among evaluation-appropriate cases, excluding those with tandem lesions or ATBI, the corresponding rates were 72.9%, 24.8%, 6.9%, and 45.5%, respectively. When analyzed by occluded vessel type, the rates for MeVO were 71.9%, 23.7%, 6.1%, and 45.7%, respectively, while those for LVO were 74.0%, 26.0%, 7.7%, and 45.1%. According to device size, the outcomes for the 2/15 mm Tron FX were 72.9%, 23.5%, 4.7%, and 50.0%, respectively, and those for the 4/20 mm device were 72.9%, 25.6%, 8.3%, and 42.5%. **Conclusions**: The PMS results for the Tron FX thrombectomy device were excellent, particularly for MeVO and the 2/15 mm model. These findings suggest that the Tron FX may help improve thrombectomy outcomes in MeVO.

## 1. Introduction

In 2015, when the efficacy of mechanical thrombectomy (MT) was first established, eligible patients included those with a computed tomography (CT)–Alberta Stroke Program Early CT Score (CT-ASPECTS) of 6 or higher and occlusion of the internal carotid artery (ICA) or the M1 segment of the middle cerebral artery (MCA) within 6 h of onset [1,2,3,4,5,6]. In 2018, two randomized controlled trials (RCTs) expanded the eligibility criteria to include patients within 24 h of onset [7,8,9,10]. In 2022, additional RCTs confirmed the efficacy of MT for large-vessel occlusion (LVO) with large ischemic regions [11,12,13,14,15,16] and for posterior circulation occlusion [17,18], suggesting further expansion of indications in the future. In contrast, MT for medium-vessel occlusion (MeVO) has not yet demonstrated efficacy, even in recent RCTs [19,20], and remains a challenge, particularly regarding the development of appropriate devices.

The Tron FX (Jimro, Gunma, Japan) is a stent retriever thrombectomy device that underwent a clinical trial in Japan in 2016. Its most distinctive feature is the availability of a 2/15 mm model designed for vessels with diameters of 1.5–2-mm, and the clinical trial results have been previously reported [21]. Regulatory approval was granted by Japan’s Pharmaceutical Affairs Agency in 2019, and postmarketing surveillance (PMS) was completed in 2020. Here, we report the PMS results for the Tron FX stent retriever, with a focus on MeVOs.

## 2. Materials and Methods

The study included data from 240 initial consecutive patients who underwent thrombectomy with Tron in the 1st pass at 24 centers (Appendix A) where Tron became available after its approval by the Japanese regulatory authorities. Patient background data included age, sex, pretreatment U.S. National Institutes of Health Stroke Scale (NIHSS) score, CT-ASPECTS, occluded vessel, and onset-to-door time (OTD). Occluded vessels were classified as the ICA, M1, M2, and M3 segments of the MCA, anterior cerebral artery (ACA), vertebral artery (VA), basilar artery (BA), or posterior cerebral artery (PCA). In addition, the presence or absence of recombinant tissue-type plasminogen activator (rt-PA), use of balloon guiding catheter and aspiration catheter and the size of the Tron, 4/20 or 2/15 mm, were registered as treatment details. Treatment outcome was evaluated by modified thrombolysis in cerebral infarction (mTICI) score ≥ 2b, first-pass effect (FPE), modified FPE (mFPE), and modified Rankin Scale score (mRS) 0–2 at 90 days. Cases with a pre-mRS score of 3 or higher were excluded from the mRS assessment. Safety endpoints were assessed for symptomatic intracranial hemorrhage (sICH) within 24 h and mortality within 90 days, with sICH defined as a worsening of the NIHSS score by 4 or more points because of intracranial hemorrhage.

To evaluate the true efficacy and safety of the Tron FX stent retriever, cases in which the device was used after the 2nd pass, and those with tandem occlusion, atherothrombotic brain infarction (ATBI), or percutaneous transluminal angioplasty (PTA) implementation were excluded. Furthermore, ICA, M1, BA, and VA occlusions were defined as LVO, and M2, M3, ACA, and PCA occlusions were defined as MeVO, and each endpoint was evaluated. Categorical variables were compared between groups using Fisher’s exact test, and continuous variables were compared using the Wilcoxon rank sum test. Similar analyses were conducted for the 4/20 mm and 2/15 mm device subgroups. Missing endpoint data were not imputed. Continuous variables were expressed as mean (standard deviation) and/or median (range), and categorical variables were presented as frequency and percentage. Statistical analyses were performed using R software version 4.4.3 (R Foundation for Statistical Computing, Vienna, Austria).

## 3. Results

A total of 244 cases in which a Tron FX stent retriever was used at participating centers from October 2019 to August 2020 were included in the analysis. Patient characteristics and treatment outcomes for all cases are summarized in Table 1. The median age was 78.5 (range 27–98) years, with 132 male patients (54.1%). The median NIHSS score was 17 (range 1–40), the median onset-to-door time was 133.5 (range 0–7200) minutes, and the median CT-ASPECTS score was 9 (range 0–10). The occluded vessels included the ICA in 19 cases (7.8%); the M1, M2, and M3 segments of the MCA in 91 (37.3%), 99 (40.6%), and 17 (7.0%) cases, respectively; and the ACA and PCA in 6 cases (2.5%) each. Intravenous rt-PA was administered in 111 cases (45.5%). A balloon-guiding catheter was used in 95.1% of cases, and an aspiration catheter was used concomitantly in 69.3%. The FPE and mFPE rates were 23.4% (57 cases) and 38.5% (94 cases), respectively. Final mTICI ≥ 2b was achieved in 70.9% (173 cases). Symptomatic intracranial hemorrhage within 24 h occurred in 6.1% (15 cases), with a favorable prognosis rate of 40.8% at 90 days and a mortality rate of 12.8%.

Factors contributing to cases that did not meet the Tron FX clinical trial eligibility criteria are shown in Table 2. To evaluate the device’s performance and safety, 15 cases in which a device other than the Tron FX was used for the first pass, 6 cases of tandem occlusion, and 6 cases of ATBI—which were likely to require additional treatment—were excluded. In the remaining cases, the FPE and mFPE rates were 24.8% and 39.0%, respectively; the final mTICI ≥ 2b rate was 72.9%; the sICH rate within 24 h was 6.9%; and 45.5% of patients achieved an mRS 0–2 at 90 days (Table 2).

Among the cases appropriate for evaluation, the occluded vessels included the M1 segment in 38.5% and the ICA in 7.3%. LVO was identified in 104 cases (47.7%), and MeVO in 114 (52.3%). MeVO comprised 76.3% M2, 14.0% M3, 4.4% ACA, and 5.3% PCA occlusions, and the 2/15 mm device was used in 69.3% of these cases. For MeVO, the FPE, mFPE, and final mTICI ≥ 2b rates were 23.7%, 41.2%, and 71.9%, respectively, compared with 26.0%, 36.5%, and 74.0%, respectively, for LVO. The sICH rate within 24 h for MeVO was 6.1%, and 45.7% of patients had a favorable prognosis at 90 days (Table 3).

Treatment results by device size are shown in Table 4. Among the 85 Tron FX 2/15 mm devices used, 68.2% were for M2 occlusions and 92.9% were for MeVO. The FPE and mFPE rates were 23.5% and 42.4%, respectively. Final mTICI ≥ 2b was achieved in 72.9% of cases, 50.0% had an mRS 0–2 at 90 days, and sICH occurred in 4.7%.

## 4. Discussion

The Tron FX is a stent retriever thrombectomy device that underwent a clinical trial in Japan from 2016 to 2017, with regulatory approval granted by Japan’s Pharmaceutical Affairs Agency in 2019. The results of that trial were previously reported in 2021 [14]. In the PMS reported here, data were available for the 4/15 mm and 2/15 mm models, and additional data for the 6/45 mm and 1.5/15 mm models are currently being collected. Unlike clinical trials, this PMS included patients with diverse clinical backgrounds and real-world treatment conditions, as summarized in Table 2. Therefore, to evaluate the true efficacy and safety of the Tron FX, we selected appropriate cases for analysis—specifically, those in which the device was used during the first pass, excluding cases of difficult recanalization such as tandem lesions and ATBI. The results, shown in Table 1, demonstrated favorable outcomes for FPE, mFPE, effective recanalization, and mRS at 90 days. Notably, the low frequency of symptomatic intracranial hemorrhage underscores the strong safety profile of the Tron FX stent retriever.

Numerous RCTs have demonstrated the efficacy of thrombectomy for occlusions involving the ICA, M1, and posterior circulation. However, even recent RCTs have not confirmed its efficacy for distal MeVO [19,20]. Previous meta-analyses [22] and large retrospective cohort studies [23] comparing M1 and M2 occlusions, and determining whether the first-line approach should be contact aspiration or stent retriever use, as well as related risk factors. These meta-analyses have reported recanalization rates of 72.8–86.9% and sICH rates was 2.6–10% [22,24,25,26]. Moreover, intravenous tissue plasminogen activator treatment alone has demonstrated relatively favorable results for MeVO [27], with early recanalization observed in more than 40% of cases—often surpassing outcomes of thrombectomy for MeVO [28]. Because the risk of sICH is generally low in this population, thrombectomy devices for MeVO must combine high safety with reliable recanalization performance. Consequently, the development of small, low-profile stent retrievers, such as the Tron FX, represents an important advancement in this field.

One of the most distinctive features of the Tron device is its 2/15 mm model, designed for target vessels less than 2 mm in diameter and compatible with a 0.0165-inch microcatheter. Few stent retrievers are designed for such small vessels with compatible microcatheters of 0.017 inches (0.43 mm) or smaller; these include the SolitaireX, Tigertriever [29,30], Catchview Mini [31,32,33], and pREset LITE [34,35]. The results obtained for the Tron 2/15 mm in this study were comparable to those previously reported for these stent retrievers, with a recanalization rate of 72.9% and an sICH rate of 4.7% (Table 5). The present findings suggest that the structure of the Tron 2/15 mm may be particularly suitable for small-diameter vessels. Because these stent retrievers have only been reported in a limited number of cases, further accumulation of treatment data will be essential for future analysis.

The present study has several limitations. First, this was a single-arm registry study, and the number of cases was relatively small. The indications for treatment and device selection were determined independently by each physician and institution. Additionally, because the dataset represents real-world data, it included some examples of off-label use. Most importantly, as this PMS relied on investigator-reported data, image and event evaluations were performed by each registrant rather than by an independent third party.

## 5. Conclusions

The results of a new stent retriever, the Tron FX thrombectomy device, were excellent, with a recanalization rate of 72.9%, an sICH rate of 6.9%, and a favorable prognosis rate of 45.5% at 90 days. The results of treatment for MeVO and the use of 2/15 mm were also favorable, suggesting potential for expanding MT indications for MeVO in the future. 

## Figures and Tables

**Table 1:** Clinical results of the Tron FX device based on postmarketing surveillance.

		Overall (*n* = 244)	Evaluation Appropriate Cases (*n* = 218)	Clinical Trial Eligible Cases (*n* = 130)
Sex (Male)		132 (54.1%)	115 (52.8%)	72 (55.4%)
Age (mean ± SD, median, range)		76.8 ± 10.8, 78.5, 27–98	77.1 ± 10.9, 79.0, 27–98	76.2 ± 10.1, 77.0, 52–98
NIHSS (mean ± SD, median, range)		17.0 ± 7.4, 17, 1–40	17.0 ± 7.3, 17, 1–40	15.8 ± 7.4, 14, 2–40
CT-ASPECTS (median, range)		9, 0–10	9, 0–10	10, 6–10
tPA use		111 (45.5%)	104 (47.7%)	70 (53.8%)
OTD (minutes; median, range)		133.5, 0–7200	133.5, 0–7200	130.5, 0–7200
Occlusion site	ICA	19 (7.8%)	16 (7.3%)	8 (6.2%)
	M1	91 (37.3%)	84 (38.5%)	42 (32.3%)
	M2	99 (40.6%)	87 (39.9%)	59 (45.4%)
	M3	17 (7.0%)	16 (7.3%)	9 (6.9%)
	ACA	6 (2.5%)	5 (2.3%)	3 (2.3%)
	VA	2 (0.8%)	1 (0.5%)	1 (0.8%)
	BA	4 (1.6%)	3 (1.4%)	3 (2.3%)
	PCA	6 (2.5%)	6 (2.8%)	5 (3.8%)
Balloon guiding catheter		232 (95.1%)	208 (95.4%)	124 (95.4%)
Aspiration catheter		169 (69.3%)	149 (68.3%)	85 (65.4%)
mTICI ≥ 2b		173 (70.9%)	159 (72.9%)	101 (77.7%)
FPE		57 (23.4%)	54 (24.8%)	36 (27.7%)
mFPE		94 (38.5%)	85 (39.0%)	56 (43.1%)
90-day mRS 0–2 *		92 (46.2%)	80 (45.5%)	68 (53.1%)
sICH within 24 h		15 (6.1%)	15 (6.9%)	5 (3.8%)
Mortality at 90 days		27 (12.8%)	26 (’11.9%)	11 (8.5%)

* excluded pre-mRS ≥ 3; Abbreviations: SD, standard deviation; NIHSS, U.S. National Institutes of Health Stroke Scale; CT-ASPECTS, Computed Tomography (CT)-Alberta Stroke Program Early CT Score; Rt-PA, recombinant tissue-type plasminogen activator; OTD, onset-to-door time; ICA, internal carotid artery; M1, M2, M3, segments of the middle cerebral artery; ACA, anterior cerebral artery; VA, vertebral artery; BA, basilar artery; PCA, posterior cerebral artery; mTICI, modified thrombolysis in cerebral infarction score; FPE, first-pass effect; mFPE, modified FPE, mRS, modified Rankin Scale score; sICH, symptomatic intracranial hemorrhage.

**Table 2:** Reasons for cases not meeting the eligibility criteria for the Tron clinical trial.

Use after 2nd pass	15 (13.2%)
Tandem occlusion	6 (5.3%)
ATBI or PTA implementation	6 (5.3%)
CT-ASPECTS ≤ 5	10 (6.9%)
DWI-ASPECTS ≤ 6	68 (59.6%)
Pre-mRS ≥ 3	42 (36.8%)

Aggregation by duplicate count. Abbreviations: ATBI, atherothrombotic brain infarction; PTA, percutaneous transluminal angioplasty; DWI, diffusion-weighted imaging.

**Table 3:** Clinical results of the Tron FX device in medium- and large-vessel occlusions.

		Medium Vessel Occlusion (*n* = 114)	Large Vessel Occlusion (*n* = 104)	*p* Value
Sex (Male)		61 (53.5%)	54 (51.9%)	0.9
Age (mean ± SD, median, range)		78.6 ± 9.3, 80, 52–98	75.5 ± 12.2, 78, 27–98	0.092
NIHSS (mean ± SD, median, range)		15.5 ± 7.3, 15, 1–32	18.7 ± 7.0, 20, 3–40	0.002
CT-ASPECTS (median, range)		9, 0–10	9, 0–10	0.5
tPA use		53 (46.5%)	51 (49.0%)	0.8
O2D (minutes, median, range)		148.5, 0–7200	117, 0–1059	0.11
Occlusion site	ICA	0 (0%)	16 (15.4%)	<0.001
	M1	0 (0%)	84 (80.8%)	
	M2	87 (76.3%)	0 (0%)	
	M3	16 (14.0%)	0 (0%)	
	ACA	5 (4.4%)	0 (0%)	
	VA	0 (0%)	1 (1.0%)	
	BA	0 (0%)	3 (2.9%)	
	PCA	6 (5.3%)	0 (0%)	
Size	2/15 mm	79 (69.3%)	6 (5.8%)	<0.001
	4/20 mm	35 (30.7%)	98 (94.2%)	
Balloon guiding catheter		109 (95.6%)	99 (95.2%)	>0.9
Aspiration catheter		67 (58.8%)	82 (78.8%)	<0.001
mTICI ≥ 2b		82 (71.9%)	77 (74.0%)	0.8
FPE		27 (23.7%)	27 (26.0%)	0.8
mFPE		47 (41.2%)	38 (36.5%)	0.5
90-day mRS 0–2 *		43 (45.7%)	37 (45.1%)	>0.9
sICH within 24 h		7 (6.1%)	8(7.7%)	0.8
Mortality at 90 days		12 (10.5%)	14 (13.5%)	0.5

* excluded pre-mRS ≥ 3; Abbreviations: SD, standard deviation.

**Table 4:** Clinical results of the Tron FX device according to 2/15 mm and 4/20 mm sizes.

Clinical Variable		Tron FX 2/15 mm (*n* = 85)	Tron FX 4/20 mm (*n* = 133)	*p* Value
Sex (male)		45 (52.9%)	70 (52.6%)	>0.9
Age (mean ± SD, median, range)		77.8 ± 10.2, 80, 52–98	76.6 ± 11.3, 78, 27–98	0.5
NIHSS (mean ± SD, median, range)		15.0 ± 7.2, 14, 1–32	18.4 ± 7.1, 19, 3–40	0.001
CT-ASPECTS (median, range)		9 (5, 10)	9 (0, 10)	0.4
Rt-PA use		39 (45.9%)	65 (48.9%)	0.7
OTD (minutes, median, range)		166, 0–7200	116, 0–1059	0.093
Occlusion site	ICA	1 (1.2%)	15 (11.3%)	<0.001
	M1	5 (5.9%)	79 (59.4%)	
	M2	58 (68.2%)	29 (21.8%)	
	M3	14 (16.5%)	2 (1.5%)	
	ACA	4 (4.7%)	1 (0.8%)	
	VA	0 (0.0%)	1 (0.8%)	
	BA	0 (0.0%)	3 (2.3%)	
	PCA	3 (3.5%)	3 (2.3%)	
Balloon guiding catheter		83 (97.6%)	125 (94.0%)	0.3
Aspiration catheter		52 (61.2%)	97 (72.9%)	0.075
mTICI ≥ 2b		62 (72.9%)	97 (72.9%)	>0.9
FPE		20 (23.5%)	34 (25.6%)	0.8
mFPE		36 (42.4%)	49 (36.8%)	0.5
90-day mRS 0–2 *		35 (50.0%)	45 (42.5%)	0.4
sICH within 24 h		4 (4.7%)	11 (8.3%)	0.4
Mortality at 90 days		9 (10.6%)	17 (12.8%)	0.7

* excluded pre-mRS ≥ 3; Abbreviations: SD, standard deviation.

**Table 5:** Stent retrievers for MeVO.

Parameter	pRESET LITE [35]	CatchView Mini [33]	Tigertriever 13 [29]	Tron FX 2/15 mm
Number of cases	227	196	45	85
Occlusion site	A1, A2, M2, M3, M4, P1, P2	A1, A2, A3, M2, M3, P1, P2, P3	A2, A3, M3, M4, P2, P3	ACA, PCA, M1, M2, M3, ICA
Final mTICI ≥ 2b	89%	94.8%	84.4%	72.9%
First-pass effect (FPE)	31%	55.1%	26.7%	23.5%
90-day mRS 0–2	58%	73.9%	53.5%	50.0%
sICH within 24 h	—	0.5%	7.0%	4.7%
Mortality at 90 days	12%	6.6%	—	10.6%

## Data Availability

The data that support the findings of this study are available from the corresponding author upon reasonable request.

## Abbreviations

The following abbreviations are used in this manuscript:

ASPECTS	Alberta Stroke Program Early CT Score
CT	computed tomography
DWI	diffusion-weighted imaging
ICA	internal carotid artery
MCA	middle cerebral artery
RCT	randomized controlled trial
MeVO	medium-vessel occlusion
PMS	postmarketing surveillance
NIHSS	U.S. National Institutes of Health Stroke Scale
OTD	onset-to-door time
M1, M2, M3	segments of the middle cerebral artery
ACA	anterior cerebral artery
VA	vertebral artery
BA	basilar artery
PCA	posterior cerebral artery
rt-PA	recombinant tissue-type plasminogen activator
mTICI	modified thrombolysis in cerebral infarction
FPE	first-pass effect
mFPE	modified first pass effect
mRS	modified Rankin Scale score
sICH	symptomatic intracranial hemorrhage
LVO	large-vessel occlusion
ATBI	atherothrombotic brain infarction
SD	standard deviation
BMT	best medical therapy (best available medical management)

## Appendix A

Principal Investigator: Nobuyuki Sakai, Kobe City Medical Center General Hospital. Naoto Kimura, Iwate Prefectural General Hospital: Masataka Takeuchi, Seisho Hospital: Hirotoshi Imamura, Kobe City Medical Center General Hospital: Yasushi Matsumoto, Kohnan Hospital: Yoshinori Akiyama, Tenri Hospital: Tsuyoshi Ohta, Kochi Health Sciences Center: Keisuke Imai, Japanese Red Cross Society Kyoto Daiichi Hospital: Tetsu Satow, National Cerebral and Cardiovascular Center: Tatsuya Ogino, Nakamura Memorial Hospital: Rei Kondo, Yamagata City Hospital Saiseikan: Kazumi Kimura, Nippon Medical School Hospital: Takahiro Ota, Tokyo Metropolitan Tama Medical Center: Masafumi Morimoto, Yokohamashintoshi Neurosurgical Hospital: Osamu Masuo, Yokohama Municipal Citizen‘s Hospital: Yukiko Enomoto, Gifu University Hospital: Shinzo Ota, Ota Memorial Hospital: Shinya Koyama, Saitama Medical University International Medical Center: Nobutaka Horie, Nagasaki University Hospital: Hiroshi Yamagami, National Hospital Organization Osaka National Hospital: Shinichi Yoshimura, Hyogo Medical University: Yuji Matsumaru, University of Tsukuba Hospital: Taketo Hatano, Kokura Memorial Hospital: Masaru Hirohata, Kurume University Hospital.
